# Integrated photonic guided metalens based on a pseudo-graded index distribution

**DOI:** 10.1038/s41598-020-58029-z

**Published:** 2020-01-24

**Authors:** Karim Hassan, Jacques-Alexandre Dallery, Pierre Brianceau, Salim Boutami

**Affiliations:** 1 Université Grenoble Alpes, CEA, LETI, Minatec Campus, F-38054 Grenoble, France; 2 Vistec Electron Beam GmbH, Jena, 07743 Germany

**Keywords:** Metamaterials, Silicon photonics, Nanophotonics and plasmonics, Sub-wavelength optics

## Abstract

In this article, we report an integrated optical nanolens exhibiting a pseudo-graded index distribution in a guided configuration. This dielectric metalens relies on a permittivity distribution through dielectric strips of the core material, which is compatible with existing silicon photonic technology. We show in this paper that effective medium theory (EMT) inaccurately predicts the focal length of such devices, and we propose an efficient and accurate design approach based on 2D finite element method (FEM) mode calculations that are in good agreement with 3D FDTD simulations. The lens was fabricated on a 200 mm silicon on insulator pilot line, and fibre-to-fibre optical characterizations revealed an excellent transmission of 85% for TM polarization, in line with the simulated performance (90%). The proposed approach can be easily extended to width-variable strips, enabling the realization of all types of graded index devices, especially those derived from transformation optics.

## Introduction

Integrated photonic components are expected to play an important role in optical communication, computing, and sensing. However, despite the high confinement factor provided by many core cladding configurations, the footprint of purely dielectric photonic devices remains significantly larger than the wavelength in the propagation direction. For instance, single-mode to multimode waveguide transitions are tens to hundreds of times longer than the optical wavelength^1^, even with optimized shapes used to shorten the tapering^2^. For purely passive functions, transformation optics (TO)^3–5^ has been proposed to generate ultra-compact devices. However, the devices obtained with TO are generally not directly compatible with CMOS monolithic integration due to the continuously variable permittivity distributions.

Phase grading and index grading are sophisticated solutions to manipulate the optical properties of a free space light beam. While some materials with suitable permittivity profiles are available to effectively generate a spatially graded index (GRIN), such as for a GRIN lens^6^, alternatives such as diffraction gratings^7,8^ or, more generally, metasurfaces^9–13^ have been proposed based on subwavelength features. A diffractive medium composed of transparent subwavelength microstructures can indeed emulate a continuously variable effective index profile^7^. Such metasurfaces can also benefit from thin subwavelength metallic resonators arranged in pseudo-arrays to obtain abrupt phase changes, drastically reducing the optical path necessary to shape light beams at the expense, however, of some metallic losses^9^.

Graded index approaches have not been widely transferred to integrated optics, despite the advantages highlighted in free space optics or in fibre optics. Recently, pseudo-graded index structures based on waveguide core thickness variations were realized using greyscale lithography^14^. Planar approaches, which are more compatible with CMOS Si photonics, have also been proposed^15–17^. These approaches exploit effective medium theory (EMT)^18^ to generate a graded index using binary structures of spatially varying filling factor^16^.

In this paper, we revisit the design of a metalens, acting as a mode converter from a multimode waveguide excited at its fundamental mode to a single-mode waveguide. We especially show that second-order EMT, although useful for designing an efficient lens, is ineffective in predicting the focal length. We propose a more accurate approach based on FEM mode calculations to efficiently determine both the lens profile and focal length, taking into account technological constraints. We successfully compare this approach with 3D-FDTD, and we fabricate and characterize the resulting structure on a 200 mm Si platform. The proposed approach is applicable to any device exhibiting a continuously graded permittivity variation, such as most devices obtained from TO^3^.

## Results and Discussion

**Figure 1:**
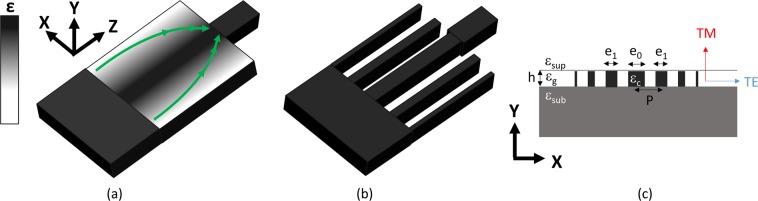
Schematic view of the integrated nanophotonic lens: (**a**) equivalent graded index lens, (**b**) 1D strip-based metalens and (**c**) section of the metalens with relevant structural and optical parameters.

The device we consider is a mode converter, represented in Fig. 1, that acts as a quarter period of a graded index beating lens, with the device length L defined as the distance from the multimode waveguide (width W) to the single-mode waveguide (width w) (Fig. 1), equivalent to the focal length. The integrated lens is composed of longitudinal parallel strips of decreasing width from the centre to the border, with the strip centres located on a periodic array of period P. We denote by ε_sub_, ε_sup_, and ε_c_, respectively, the permittivities of the substrate, the superstrate (also assumed in the gaps between strips), and the strips (core waveguide material). We will design the lens taking into account the technological limits for the pseudo-graded index, i.e., the maximum/minimum filling factor of the core material f_max_/f_min_. The transverse variation in the strip width generates a phase delay decreasing from the centre to the border of the lens, thus producing focalization^7–13^. Such a lens has to exhibit an effective index profile, as derived from fibre optics^19–21^, satisfying:

1 $${n}_{eff}^{2}\left(x\right)={n}_{eff}^{2}\left(0\right).sec{h}^{2}\left(\frac{\pi }{2L}x\right)$$

As we are in a planar guided configuration, radiative losses must be avoided, which, in addition to the filling factor technological limits, adds the supplementary constraint that the effective index has to be greater than both the substrate and superstrate refractive indices, which results in:

2 $$Max\left({n}_{eff}^{2}\left({f}_{\min }\right),Max\left({\varepsilon }_{sub},{\varepsilon }_{sup}\right)\right) < {n}_{eff}^{2}\left(f\right) < {n}_{eff}^{2}\left({f}_{max}\right)$$

From (1) and (2), we can deduce that the operating length L of the device, given technological constraints, is:

3 $$L=\frac{\pi W}{4}{\left[log\left(\frac{{n}_{eff}({f}_{max})+\sqrt{{{n}_{eff}}^{2}({f}_{max})-Max({n}_{eff}^{2}({f}_{min}),Max({\varepsilon }_{sub},{\varepsilon }_{sup}))}}{{n}_{eff}({f}_{min})}\right)\right]}^{-1}$$

One has to distinguish the two polarization cases: TE (electric field parallel to the substrate interface) and TM (magnetic field parallel to the substrate interface). The common approach for evaluating ${n}_{eff}$ for gratings has been described by Rytov^18^ and consists of expressing the EMT transcendental equations as a polynomial series. While the zeroth-order approximation of these equations is fairly accurate for very small periods compared to the wavelength, the second-order approximation, known as second-order EMT, is more commonly used because it accounts for a (*P*/*λ*)^2^ term that provides more accurate results^18,22,23^. According to second-order EMT:

4 $$\begin{array}{ccc}{\mathop{{\rm{n}}}\limits^{\sim}}_{eff-TE-EMT}^{2}(f) & = & \frac{f}{{n}_{slab-TE}^{2}}+\frac{1-f}{{\varepsilon }_{g}}+\frac{1}{3}\left[\frac{P}{\lambda }\pi \,f(1-f)\left(\frac{1}{{n}_{slab-TE}^{2}}-\frac{1}{{\varepsilon }_{g}}\right)\right.\\  &  & \left.\sqrt{f\cdot {n}_{slab-TE}^{2}+(1-f){\varepsilon }_{g}}{\left(\frac{f}{{n}_{slab-TE}^{2}}+\frac{1-f}{{\varepsilon }_{g}}\right)}^{3/2}\right]^{2}\end{array}$$

5 $${\mathop{{\rm{n}}}\limits^{ \sim }}_{eff-TM-EMT}^{2}(f)=f\cdot {n}_{slab-TM}^{2}+(1-f){\varepsilon }_{g}+\frac{1}{3}{\left[\frac{P}{\lambda },\pi ,f,(,1,-,f,),(,{n}_{slab-TM}^{2},-,{\varepsilon }_{g},)\right]}^{2}$$

where ${n}_{slab-TE}$ and ${n}_{slab-TM}$ are the effective indices of the slab without corrugation, which are the analytical solutions of the equations^24^:

6 $$\left\{\begin{array}{c}\frac{2\pi }{\lambda }\sqrt{{\varepsilon }_{c}}\sqrt{\frac{{\varepsilon }_{c}-{n}_{slab-TE}^{2}}{{\varepsilon }_{c}}}h-{\rm{a}}{\rm{t}}{\rm{a}}{\rm{n}}\left(\sqrt{\frac{{n}_{slab-TE}^{2}-{\varepsilon }_{sub}}{{\varepsilon }_{c}-{n}_{slab-TE}^{2}}}\right)-{\rm{a}}{\rm{t}}{\rm{a}}{\rm{n}}\left(\sqrt{\frac{{n}_{slab-TE}^{2}-{\varepsilon }_{sup}}{{\varepsilon }_{c}-{n}_{slab-TE}^{2}}}\right)=0\\ \frac{2\pi }{\lambda }\sqrt{{\varepsilon }_{c}}\sqrt{\frac{{\varepsilon }_{c}-{n}_{slab-TM}^{2}}{{\varepsilon }_{c}}}h-{\rm{a}}{\rm{t}}{\rm{a}}{\rm{n}}\left(\frac{{\varepsilon }_{c}}{{\varepsilon }_{sub}},\sqrt{\frac{{n}_{slab-TM}^{2}-{\varepsilon }_{sub}}{{\varepsilon }_{c}-{n}_{slab-TM}^{2}}}\right)-{\rm{a}}{\rm{t}}{\rm{a}}{\rm{n}}\left(\frac{{\varepsilon }_{c}}{{\varepsilon }_{sup}},\sqrt{\frac{{n}_{slab-TM}^{2}-{\varepsilon }_{sup}}{{\varepsilon }_{c}-{n}_{slab-TM}^{2}}}\right)=0\end{array}\right.$$

**Figure 2:**
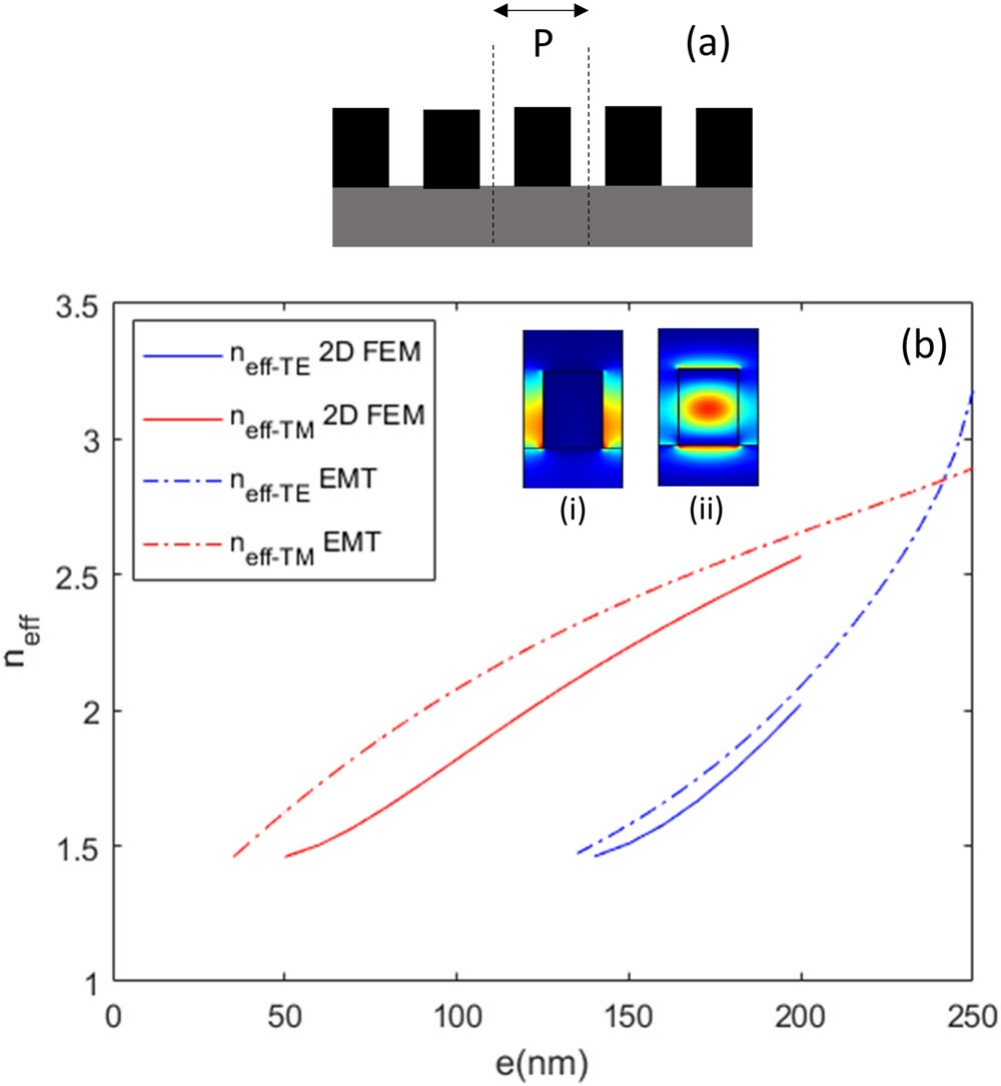
Design of the metalens, at λ = 1.31 µm, for W = 3 µm, w = 0.35 µm, h = 0.31 µm, P = 250 nm, extreme strip widths e_0_ = 200 nm and e_6_ = 50 nm, ε_c_ = 3.506 (Si), ε_sub_ = 1.4468 (Si02), ε_g_ = 1, and ε_sup_ = 1. (**a**) Geometry of FEM mode effective index calculation, considering a periodic array of strips with lateral periodic conditions (dashed lines) and vertical PMLs. (**b**) Effective index, as a function of strip width e, of the TE mode (blue lines) and the TM mode (red lines), calculated by FEM (solid lines) or second-order EMT (dashed-dotted lines). Inset: (i) Ex field of the TE mode, and (ii) Ey field of the TM mode for a width e = 150 nm.

We will compare the EMT effective index approach given by Eqs. (4–6) with an approach based on FEM, consisting of calculating the effective index of the mode in a periodic array of identical strips (Fig. 2(a)). This comparison is performed for a realistic silicon on insulator (SOI) metalens operating at a wavelength of λ = 1310 nm. The silicon thickness is h = 310 nm, and we fix our minimum feature size to be 50 nm, i.e., the filling factor is between f_min_ = 0.2 and f_max_ = 0.8 for a subwavelength period P = 250 nm. The multimode waveguide width is chosen to be W = 3 µm (for low loss photonic circuitry^25^) and that of the single-mode waveguide to be w = 350 nm, in line with standardized values for strip waveguides with a 310 nm-thick SOI platform. The refractive indices used are from the literature^26^ and ellipsometry measurements.

The comparison in Fig. 2(b) shows that even if the EMT effective index profile evolution looks broadly correct, there is some bias in the effective index values, especially for TM polarization. Table 1 presents the strip widths that satisfy Eqs. (1) and (2) and the operating lengths given by Eq. (3) for TE polarization based on the FEM effective index calculations of Fig. 2 and for TM polarization based on both the EMT and FEM effective index calculations of Fig. 2. TM polarization represents the most interesting case because it allows a wider variation of the filling factor while maintaining guiding in the slab (Eq. 2). Although the strip widths predicted by FEM and EMT for TM are similar, the operation length predicted by EMT is 25% greater than that by FEM, which represents a significant difference (Table 1).

**Table 1:** Values of strip widths used to design the metalens and corresponding estimated operating lengths, deduced from the FEM TE design, FEM TM design, and EMT TM design.

L (µm)	e_0_ (nm)	e_1_ (nm)	e_2_ (nm)	e_3_ (nm)	e_4_ (nm)	e_5_ (nm)	e_6_ (nm)
L_TE-FEM_ = 2.725	200	199	194	186	176	162	136
L_TM-FEM_ = 2.022	200	192	172	144	115	87	50
L_TM-EMT_ = 2.193	200	191	166	134	102	74	50

**Figure 3:**
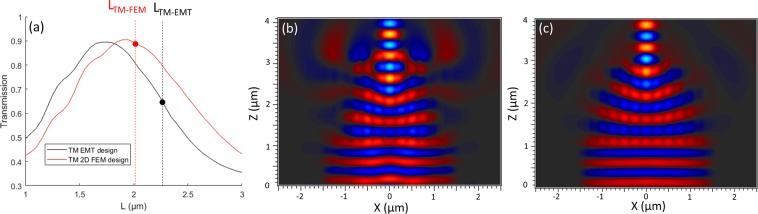
3D-FDTD simulation of FEM and EMT designs for TM injection. (**a**) FDTD-calculated transmission as a function of device length for the TM-EMT design (black solid line) and the TM-FEM design (red solid line). Dashed lines indicate the optimal lengths estimated by both the TM-EMT and TM-FEM methods for the corresponding designs, leading to the operating points indicated by the black and red circles, respectively. Real part of Ey at mid-height of the membrane, at the estimated optimal operating points, for (**b**) the TM-EMT design and (**c**) the TM-FEM design (λ = 1.31 µm).

For confirmation using a numerical method, we performed 3D-FDTD simulations of the designs optimized using TM EMT and FEM with a uniform calculation grid of 15 nm for the three axes and perfectly matched layers as boundary conditions. As expected, both designs perform efficient mode conversion (90%); however, only the FEM design performs the conversion at the expected device length. The EMT design conversion is only 65% at its expected operating length (Fig. 3).

## Experiments and Methods

An ultra-compact metalens was fabricated on an SOI substrate using 200 mm CMOS pilot line processing tools for TM operation, following the FEM optimized design. Dedicated lithography and etching processes were developed specifically for this device since the filling factors of the line/trenches calculated analytically resulted in a relatively high aspect ratio (~6) to make the device compact.

Starting from the 310 nm-thick silicon top layer of the 200 mm SOI wafer with a buried oxide layer of 1 µm, a triple layer of resist and hard mask was deposited. The electron-beam lithography process (VISTEC variable-shape VB6B) was optimized using 85 nm of negative tone resist from TOK (OEBR-CAN038) on top of 30 nm of silicon antireflective coating (SiARC) ISX412 and 130 nm of spin on carbon (SOC) HM8102. The combination of these three layers allowed us to define elements with features down to 18 nm (lines) on the resist, which were subsequently etched using a HBr-based reactive ion. The trilayer stack was etched in a single sequence with an OES (optical emission spectroscopy) monitoring system to adjust the etching time of each layer (20” for the SiARC, 38” for the SOC, 4” to remove any natural oxide on the SOI, and 105” for the 310 nm silicon, including 5 seconds of overetching to achieve clean right angles at the bottom of the patterns (waveguides and lines)).

**Figure 4:**
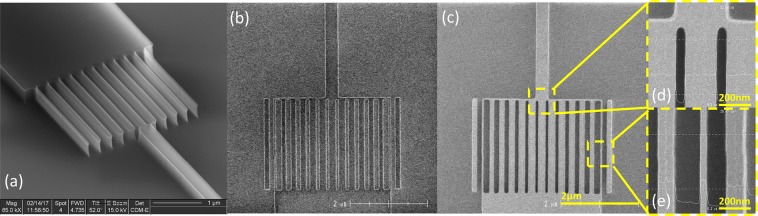
SEM images of the fabricated metalens: (**a**) metalens fabricated without lateral sustaining strips, (**b**) resist after lithography with lateral sustaining strips, (**c**) final metalens fabricated with lateral sustaining strips, (**d**) zoomed-in view of the maximal filling factor region, and (**e**) zoomed-in view of the minimal filling factor region. The strips widths from the centre to border are 200 nm, 180 nm, 170 nm, 155 nm, 140 nm, 110 nm, and 40 nm.

However, after etching, we observed that the external 37 nm-wide lines were not present (Fig. 4(a)). To rectify this problem, we repeated the process, adding two supplementary lateral lines on the sides of the device and one on the output of the GRIN area (Fig. 4(b)), to achieve a homogeneous energy density over the entire device during lithography. Figure 4(c–e) show top view SEM images of the silicon device, with strip widths from the centre to the border of the metalens ranging from 200 ± 5 nm to 40 ± 5 nm, measured with a calibrated 200 mm pilot line SEM tool (HCG4000). The obtained strip widths are slightly different from the target values, which could be improved using optical proximity correction (OPC).

**Figure 5:**
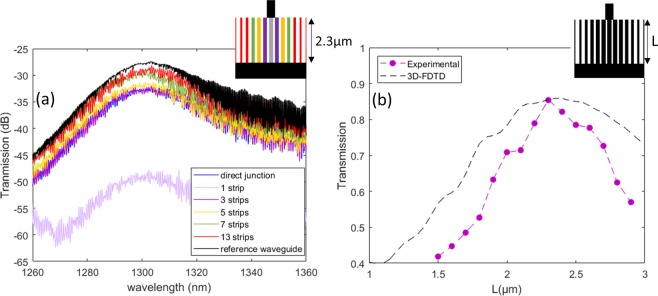
(**a**) For the optimal experimental device length L_exp_ = 2.3 µm, transmission spectra as a function of the total number of strips compared to an abrupt transition and a reference waveguide. (**b**) Device transmission at λ = 1.31 µm as a function of device length L.

Fibre-to-fibre characterization was performed to measure the transmission efficiency of this compact GRIN lens using 200 mm probing stations. The experimental setup for O-band tests included a tuneable laser source (Tunics T100S from Yenista) and its associated photodetector (CT400), providing a picometer resolution in the 1260–1360 nm range. Cleaved fibres were positioned with a self-alignment routine over fibre grating couplers, and the TM polarization was controlled with a synthesizer from Agilent. Several metalenses of various lengths were tested, including an input waveguide coupler, a 200 µm-long linear transition to a 3 µm width waveguide, the metalens, a 350 nm-wide waveguide, and an output grating coupler. The reference measurement was achieved using two waveguides: one with the two opposite linear transitions and one with the straight 350 nm waveguide. The average of both gives the reference measurement accounting for all losses except that of the metalens. The resulting transmission spectra are plotted in Fig. 5(a) for a metalens of L = 2.3 µm as a function of the number of strips fabricated. As expected, central strips are more relevant to the focalization effect, as the energy density of the waveguide mode is maximal at the centre and decreasing towards the edges. For the pseudo-GRIN lens including all strips, a maximum transmission of 85% is observed (−0.6 dB), in line with the FDTD simulation (90%). As a matter of comparison, an extra measurement was performed with direct butt coupling of the multimode waveguide to the single-mode waveguide, with a high insertion loss of −5 dB, confirming the utility of the metalens. With respect to design rules, the lens shows very little chromaticity (in the O-band), with the spectral envelope being similar for all lenses, which is related mainly to the grating coupler transmission spectrum. In Fig. 5(b), we plot the experimental transmission at λ = 1.31 µm in TM, normalized to the reference, as a function of device length. We see that the optimal length is within 15% of that predicted by the FEM model, and the shift can be confirmed using 3D-FDTD, taking into account the exact observed strip widths of the structures.

## Conclusion

In this paper, we have demonstrated an integrated metalens enabling mode conversion between the fundamental mode of a multimode waveguide and that of a single-mode waveguide, fabricated using industrial manufacturing tools on large-scale wafers.

We have shown that for such pseudo-graded index devices with realistic feature dimensions, second-order EMT can be inaccurate in generating the correct effective index profile. This was evidenced by a 25% overestimation of the optimal length of the pseudo-graded metalens. We have proposed an approach based on FEM mode calculation to improve the quantitative accuracy of the simulation, making design uncertainty negligible compared to the experimentally observed fabrication variations.

We note that the proposed design approach will not lead to devices as efficient as those obtained from optimization techniques^27^, in particular those that use level sets^28^ to impose binary structures. Our approach does not take into account input/output mode profile matching, nor can it be used to design multispectral devices. However, the proposed design approach is exclusively 2D, therefore requiring less computational resources than other design techniques, such as 3D-FDTD. It is particularly suited to the practical realization of any continuously variable refractive index region, such as those traditionally obtained from transformation optics, and can be extended to refractive index profiles that vary in the propagation direction by varying the strip width along the propagation direction. We believe that this work will pave the way towards more widespread implementation of dense TO-based integrated circuits.

## Data Availability

The datasets generated and analysed during the current study are available from the corresponding author on reasonable request.
