# Publisher Correction: Integrated photonic guided metalens based on a pseudo-graded index distribution

**DOI:** 10.1038/s41598-020-60650-x

**Published:** 2020-02-25

**Authors:** Karim Hassan, Jacques-Alexandre Dallery, Pierre Brianceau, Salim Boutami

**Affiliations:** 1 Université Grenoble Alpes, CEA, LETI, Minatec Campus, F-38054 Grenoble, France; 2 Vistec Electron Beam GmbH, Jena, 07743 Germany

Correction to: *Scientific Reports* 10.1038/s41598-020-58029-z, published online 24 January 2020

This Article contains an error in Equation 5.

$${\mathop{{\rm{n}}}\limits^{ \sim }}_{eff-TM-EMT}^{2}(f)=f\cdot {n}_{slab-TM}^{2}+(1-f){\varepsilon }_{g}+\frac{1}{3}{[\frac{P}{\lambda },\pi ,f,(,1,-,f,),(,{n}_{slab-TM}^{2},-{\varepsilon }_{g},)]}^{2}$$

should read:

$${\tilde{{\rm{n}}}}_{eff-TM-EMT}^{2}(f)=f\cdot {n}_{slab-TM}^{2}+(1-f){\varepsilon }_{g}+\frac{1}{3}{[\frac{P}{\lambda }\pi f(1-f)({n}_{slab-Tm}^{2}-{\varepsilon }_{g})]}^{2}$$

In addition, this Article contains an error in the Methods.

“The electron-beam lithography process (VISTEC variable-shape VB6B) was optimized using 85 nm of negative tone resist from TOK (OEBR-CAN038) on top of 30 nm of silicon antireflective coating (SiARC) ISX412 and 130 nm of spin on carbon (SOC) HM8102.”

should read:

“The electron-beam lithography process (VISTEC variable-shape SB3054) was optimized using 85 nm of negative tone resist from TOK (OEBR-CAN038) on top of 30 nm of silicon antireflective coating (SiARC) ISX412 and 130 nm of spin on carbon (SOC) HM8102.”

